# Perspectives of Cosmesis following Breast Conservation for Multifocal and Multicentric Breast Cancers

**DOI:** 10.1155/2015/126793

**Published:** 2015-10-04

**Authors:** Mona P. Tan, Nadya Y. Sitoh, Yih-Yiow Sitoh

**Affiliations:** ^1^MammoCare, 38 Irrawaddy Road, No. 06-21, Singapore 329563; ^2^Yong Loo Lin School of Medicine, National University of Singapore, Lower Kent Ridge Road, Singapore 119260

## Abstract

*Background.* Contemporary data suggest that breast conservation treatment (BCT) for multifocal and multicentric breast cancer (MFMCBC) may be appropriate with noninferior local control rates. However, there is a paucity of data to evaluate patient's satisfaction with cosmetic outcomes after BCT for MFMCBC. This study was performed to bridge this information gap. *Methods.* All patients treated at the authors' healthcare facility were included in the study. Patients with MFMCBC who were assessed to be eligible for BCT underwent tumour resection using standard surgical techniques with direct parenchymal closure through a single incision. After at least three years of follow-up, they were invited to participate in a survey regarding their cosmetic outcomes. *Results.* Of a total of 160 patients, 40 had MFMCBC, of whom 34 (85%) underwent successful BCT. Five-year cancer-specific survival and disease-free survival were 95.7%. Twenty of the 34 patients responded to the survey. No patient rated her cosmetic outcome as “poor.” Analysis indicated low agreement between patients' self-assessment and clinician-directed evaluation of aesthetic results. *Conclusion.* BCT for MFMCBC is feasible with acceptable survival and cosmetic outcomes. However, there appears to be a disparity between patient and clinician-directed evaluation of cosmetic results which warrant further research.

## 1. Introduction

Multifocal and multicentric breast cancers (MFMCBC) pose a formidable challenge to breast conservation treatment (BCT). Early studies of MFMCBC showed poorer local control with BCT than mastectomy [1, 2], which led to multiple ipsilateral cancers being viewed as a contraindication to BCT. However, more recent studies have shown that local control is independent of the type of surgical treatment and there is emerging data to show that BCT is a reasonable treatment option [3–5]. With the expected larger resection volumes for MFMCBC, achieving reasonable cosmesis becomes a matter of concern. Oncoplastic breast surgery (OBS) approaches have been suggested as a means to overcome potential deformities [6], but women with multicentric disease are often excluded in studies on OBS [7]. As a result, there is little information in contemporary medical literature on cosmetic outcomes and patient satisfaction following BCT for MFMCBC.

One reason for this scarcity could be the absence of standardised methods for the surgical treatment of MFMCBC. Multifocal lesions may be tackled as an extended unifocal resection. Although previously viewed as an indication for mastectomy, it has now been suggested that multicentric disease may be treated by two separate lumpectomies [8]. However, these may contravene guidelines which recommend that multiple lesions which cannot be excised through a single incision be subject to mastectomy [9]. A novel classification for MFMCBC, together with an algorithm for surgical approach, has been proposed to allow resection of MFMCBC through a single incision with minimal compromise of cosmesis [10]. Through its application, guidelines are not violated and the stringent requirements of clear margins with “no tumour on ink” [11] and acceptable aesthetics can be achieved. Using these techniques uniformly, successful BCT may be achieved for patients with MFMCBC. Since there is at present a paucity of data on cosmetic results following BCT for MFMCBC, the aim of this study was to provide some information on the feasibility of achieving BCT for MFMCBC using the segment classification and patient's perception of their aesthetic outcomes, leading to insights for future directions for treatment.

## 2. Methods

All patients treated for MFMCBC at the authors' private facility from January 2009 to December 2011 were included in this retrospective study. Patients were considered to have MFMCBC on the basis of the combined preoperative assessment with mammogram, ultrasound studies, and clinical and histologic examination. Preoperative MRI and PET scans were not routinely performed. Percutaneous biopsy of suspicious lesions was performed and if neoadjuvant chemotherapy was recommended, radioopaque clips were placed within the lesions. For clinical scenarios where percutaneous biopsy was not feasible, localisation and surgical biopsy was performed.

Eligibility for BCT was made based on the attending surgeon's assessment of the clinical indicators. For those considered to have doubtful or borderline eligibility, a recommendation was made for a “trial of BCT,” with the understanding that a mastectomy would be performed if the attempt at BCT resulted in loss of tissue that could not be repaired using volume displacement techniques without mammoplasty or contralateral symmetrisation. T4 tumours were not considered a contraindication to breast conservation [12], and if involved skin and/or muscle could be excised with anticipation of a reasonable cosmetic outcome, the patient would be offered a trial of BCT. As part of the attempt at BCT, neoadjuvant chemotherapy would be administered for downstaging if deemed appropriate.

Successful BCT for MFMCBC was defined as resection of all tumour points with negative margins, “no tumour on ink.” Intraoperative frozen section analysis was performed for all patients, both for margins and for sentinel lymph node biopsy. Those who underwent successful BCT were invited to participate in a survey regarding their cosmetic outcomes. These patients underwent repair of their tumour cavity solely through volume displacement techniques, using direct parenchymal apposition. None had mammoplasty, mastopexy, or volume replacement techniques. They were asked to rate their outcomes on a 5-point scale: 5: excellent, 4: good, 3: acceptable, 2: mediocre, and 1: poor. They were also asked if they would consider further revision surgery and to give comments on their experience and treatment satisfaction. The attending surgeon likewise listed the clinician's assessment of the final cosmetic outcome.

Statistical analyses were performed using SPSS (Chicago, IL) version 19 advanced statistical software module. Comparisons of variables were performed using the chi-squared test, Mann-Whitney* U* test, and kappa statistic where appropriate, with the log-rank test used for survival analysis.

## 3. Results

A total of 41 patients were diagnosed to have MFMCBC during the study period. However, one patient did not complete treatment and was excluded, leaving 40 patients for analysis. Of these, 35 were offered a trial of BCT and 34 (85%) underwent successful BCT. The clinicopathologic details of the patient cohort are given in Table 1. The mean age for the study cohort was 46 years and the mean pathologic size of the largest lesion was 19.6 mm. Seventy percent of the patients were Chinese. The majority presented with at least one palpable tumour.

**Figure 1:**
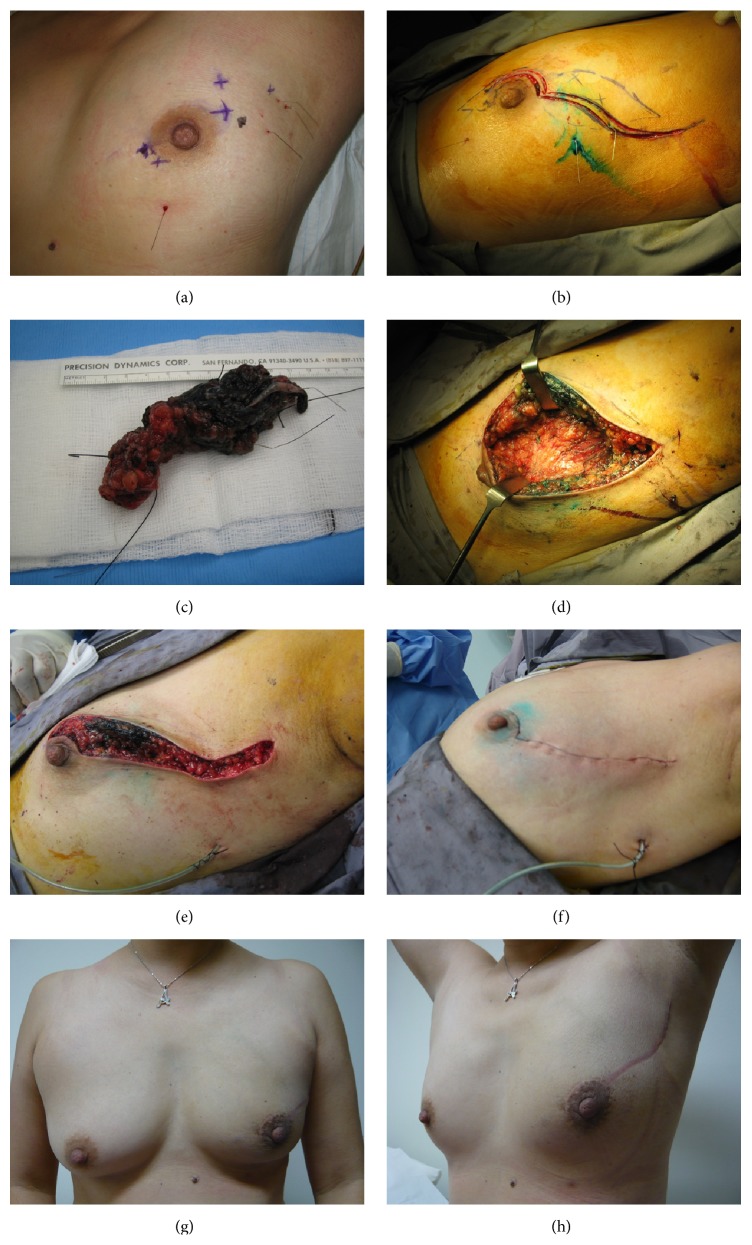
This patient was diagnosed with multicentric breast cancer at another tertiary oncology centre and offered mastectomy, which she declined. Having undergone neoadjuvant chemotherapy, (a) shows her preoperative status with multiple localisation wires in various directions. A modified boomerang incision [30] was used with a dual-pronged segment resection joined centrally (dotted lines) (b). This approach allows en bloc resection for lesions in opposite quadrants across the nipple-areolar complex through a single incision. After extirpation of all identified residual lesions, parenchymal pillars were mobilised, followed by their direct apposition with sutures (e). Her cosmetic outcome two years after completion of treatment is shown in (g) and (h). She is currently disease-free more than five years after treatment.

A segment classification for MFMCBC previously proposed was used to allow surgical planning for BCT for this cohort of patients [10]. All patients who underwent BCT had only volume displacement techniques performed with direct apposition of parenchymal walls. An example of such a procedure for a patient with multicentric disease is illustrated in Figures 1(a)–1(h). None had mammoplasty, contralateral symmetrisation, or volume replacement with implants or flaps. Of the six who underwent mastectomy, three did not choose to have reconstruction, two underwent immediate reconstruction, and one elected to have contralateral prophylactic mastectomy with bilateral reconstruction.

**Figure 2:**
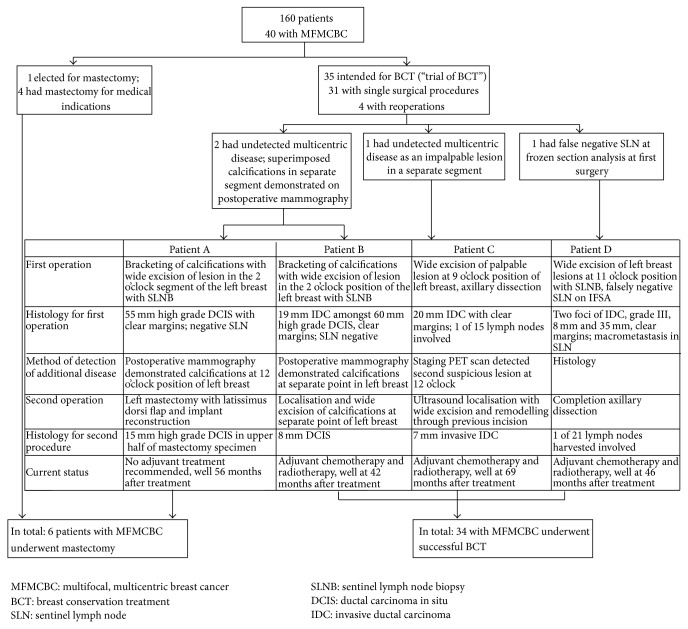
The figure summarises the clinical events surrounding reoperations among patients with multifocal and multicentric breast cancer in this study.

Four patients required repeat operations. One had an inadvertently missed second lesion which was identified on staging investigations with a PET scan. Two patients were found to have additional microcalcifications in a different segment after excision of extensive lesions, indistinguishable because of superimposition of smaller clusters onto microcalcifications exceeding 55 mm. One patient opted to undergo a mastectomy with reconstruction for a residual 15 mm ductal carcinoma in situ (DCIS), while the other underwent wide excision of a second malignant lesion leading to successful BCT. Margins were negative for both first and second procedures for all three patients. The fourth patient had multifocal cancer in the upper inner quadrant of the left breast. Margins were negative at the primary surgery but sentinel lymph node was falsely negative at frozen section analysis. As her surgery was performed in 2009, she returned to the operating room for axillary dissection. All four patients are currently well without any recurrence. Their events are summarised in Figure 2.

For the entire study cohort, five-year breast cancer-specific survival and disease-free survival were 95.7% and 92.7%, respectively. Mean time to recurrence for multifocal disease and multicentric tumours was 74.1 months and 70.2 months, respectively. Although there was a trend towards higher local control and survival for multifocal disease when compared with multicentric disease as reported in one other study [4], this did not reach statistical significance in our analysis.

**Figure 3:**
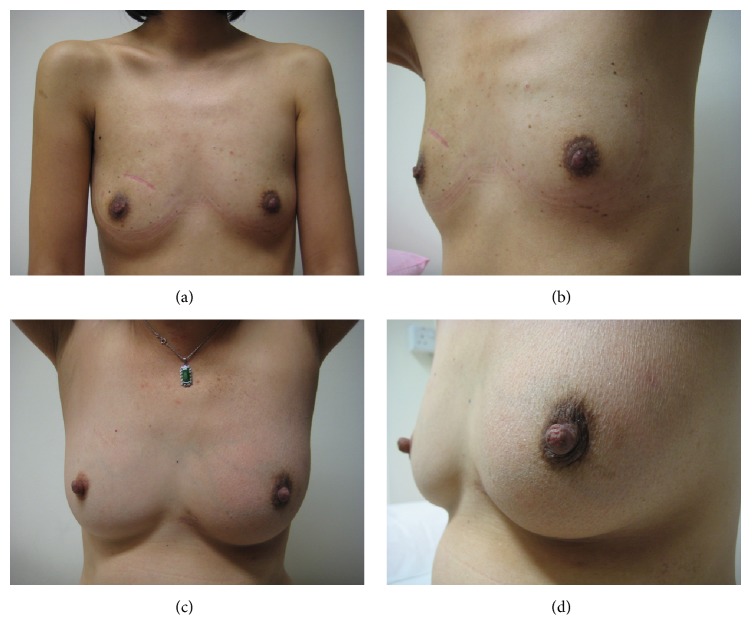
These two patients rated their cosmetic outcome differently from the surgeon's assessment. The patient shown in (a) and (b), who had two separate disease foci in the upper inner quadrant of the right breast, rated her outcome as “good” while her surgeon felt it was “excellent.” The patient depicted in (c) and (d) was treated for two clusters of microcalcifications (DCIS) in the retroareolar region. Initially offered a mastectomy at another oncology centre, she rated her final breast conservation treatment result as “satisfactory,” while her surgeon thought it was excellent.

**Figure 4:**
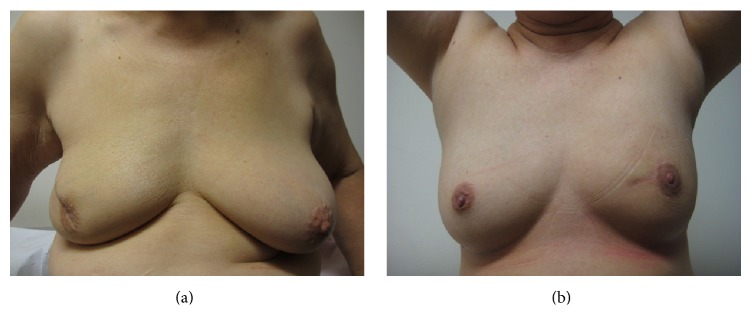
Both these patients rated their outcome in agreement with the surgeon's assessment. The patient in (a) had multifocal disease in the upper outer quadrant of the right breast associated with nipple discharge. Both she and her surgeon considered her outcome “satisfactory.” The patient whose result is shown in (b) was treated for multifocal disease, separate 14 and 18 mm grade 3 invasive ductal carcinomata in the upper inner quadrant of the left breast. She is currently well 66 months after treatment. Both she and her surgeon considered her outcome “excellent.”

Of the patients who underwent successful BCT, 20 of 34 patients agreed to participate in a survey of their cosmetic outcomes, giving a response rate of 58.8%. All patients except one patient in the cohort underwent radiotherapy. All rated their outcomes as satisfactory and above, suggesting that, in this cohort, radiotherapy did not significantly affect patient's perception of cosmetic outcome. The single patient who elected against radiotherapy rated her outcome as “satisfactory.” This patient is currently recurrence-free. Thirty-two patients with MFMCBC received chemotherapy. Twenty-eight patients had hormone-receptor positive tumours and were given endocrine therapy. Of the respondents with radiotherapy, chemotherapy, and/or endocrine treatment, none rated their outcomes as mediocre or poor. The attending clinician's outcome varied with the patient's assessment in a moderate proportion of cases (Table 2). Analysis showed that there was a relatively low level of agreement between patients' and clinician's assessment of cosmetic outcome (kappa 0.11) (Figures 3 and 4). No significant difference was noted in patient's assessment of cosmetic outcome based on hormone-receptor status and endocrine therapy (*p* = 0.24). Three patients, who rated their outcomes as “excellent,” volunteered that other clinicians had also rated their aesthetic results highly. In the process of asking patients for additional comments, a few requested viewing photographs of outcomes for others. Having seen other illustrated outcomes in pictures, some were inclined to alter their personal ratings to a higher level. However, as this was an unexpected finding, any change in rating after viewing results from other patients was not included for purposes of analysis. Only one respondent underwent nipple reconstruction but none of the others desired further surgery for correction or contralateral symmetrisation. Those who viewed symmetrisation procedures did not change their opinion on the need for further operations.

## 4. Discussion

Two recent studies provide evidence that patient's perception of body image and cosmesis may not correspond to either clinician's assessment or analysis by a computer programme [13, 14]. In the study by Dr. Santos et al., it was observed that a higher proportion of patients rated their cosmetic outcomes as “excellent” when compared to physicians' assessment or BCCT.core software. Similar findings were noted in the study by Dr. Kim and his colleagues. Results from the present study on patients with BCT for MFMCBC, although performed on a smaller scale, also mirror prior findings on the discrepancies between patient and clinician perceptions on cosmetic outcomes.

The use of BCT for MFMCBC poses a significant challenge towards achieving acceptable cosmesis. There is little in current medical literature on surgical techniques which may be consistently applied for this specific purpose. A few authors advocate the use of oncoplastic manoeuvres with mammoplasty techniques and contralateral symmetrisation where indicated [6, 15]. However, there is a paucity of information detailing the treatment of MFMCBC without the routine use of mammoplasty, wise pattern, or split reduction procedures. Self-reported impressions of cosmetic outcomes by patients are also lacking when extreme oncoplastic techniques are described [7, 15]. It has been demonstrated that patients' self-reported body image scores are directly correlated with quality of life after breast cancer treatment [14]. Eichler et al. provided some evidence that a higher proportion of women with standard lumpectomy were satisfied with the appearance of their scar than those who had mastopexy [16]. In addition, shorter operating times were associated with lumpectomy. Complication rates with standard BCT with full thickness closure compare favourably (5.2%) [17] with oncoplastic techniques with mammoplasty and contralateral symmetrisation (30–34.2%) [18, 19]. The occurrence of postoperative complications and postoperative breast asymmetry were found to be associated with higher levels of patient dissatisfaction and distrust of surgeons [20]. Hence, breast cancer treatment should be directed at avoiding these potential outcomes. Currently available data suggests that standard BCT with full thickness closure should be the treatment of choice for both unifocal cancers and MFMCBC. Its sole use in this study cohort resulted in disease-free survival similar to that of larger studies [4, 21]. Mammoplasty and contralateral symmetrisation may be reserved for a group of highly selected patients with very large tumours [15].

There is emerging evidence to demonstrate a discrepancy in patient, clinician, and software based assessment of cosmetic outcomes. Although achieving symmetry is a major concern for clinicians, it may be less of a concern for patients [14]. From the patient's perspective, there may be a difference between an acceptable breast form without severe deformity and symmetry. In future studies, perhaps a clear distinction should be made between breast form and symmetry, rather than assessing them as a single entity. Anecdotal reports have been made regarding patient's disinclination for contralateral symmetrisation procedures, in favour of a mild to moderate difference in breast tissue volumes [22, 23]. This aspect of patient's perception and satisfaction may be further researched. Self-reported cosmetic outcomes and body image score (BIS) are significant end-points for breast cancer treatment as they play an important role in patient treatment experience and long-term quality of life [14, 20]. Future treatment and its impact therefore should be directed towards improving patient's BIS.

This study was limited by its small sample size from a single centre. In addition, there may be patient response-related bias. Nevertheless, it is one of the first to provide information on patient's perception of their cosmetic outcomes after BCT for MFMCBC. There appeared to be a tendency for patients in this study cohort to alter their own cosmetic outcome scores after seeing photographs of poor outcomes. Those who were shown mammoplasty and symmetrisation procedures did not indicate any desire for further operative intervention. These were unexpected findings in the course of this study and were not accounted for in the original study design. As such, these factors could not be taken into account for analysis in the current study and could be a matter for future research. Since the majority of patients only have a single experience with breast cancer treatment, their perception and expectations of cosmetic outcomes may be unrealistic. A possible method to assist with realistic expectations in the context of decision-making may be to use pictures and charts to illustrate the options for surgical treatment, potential cosmetic outcomes, and complications for each procedure.

Over the last few decades, there has been a paradigm shift for surgical procedures in general towards minimally invasive techniques [24]. For breast surgery, contemporary data suggests that BCT is associated with higher survival rates compared with mastectomy [25–27]. In addition, standard BCT has been shown to be less frequently associated with complications, like surgical site infections, than oncoplasty, mastectomy, with or without immediate reconstruction [28, 29]. There is also emerging evidence suggesting that BCT is a reasonable option for MFMCBC [3–6]. Using standard BCT techniques for MFMCBC can offer noninferior survival outcomes in combination with lower complications, reducing iatrogenic impact and potentially increasing patient satisfaction with treatment processes [20, 24]. As self-reported body image scores and symmetry correlate with positive treatment experience and quality of life, future research is necessary to establish appropriate predictors and measures of patient satisfaction with cosmetic outcomes and direct therapy accordingly.

## 5. Conclusion

Successful BCT for MFMCBC with at least a satisfactory cosmetic outcome is feasible. Although all of the patients in this cohort rated their cosmetic outcomes as either “satisfactory,” “good,” or “excellent,” with none rating their appearance as “poor,” the results from this study suggest a low level of agreement between patient's self-assessment of cosmetic outcomes and clinician's evaluation for BCT for MFMCBC. Further research is warranted to assess correlates of cosmetic outcomes in terms of breast form, symmetry with contralateral breast, patient's satisfaction with treatment processes, and quality of life.

## Figures and Tables

**Table 1:** Summary of demographic, clinicopathologic, and outcome data for study population.

Clinicopathologic characteristic	MFMCBC (*n* = 40)		Multifocal (MF)		Multicentric (MC)		*p* value
		(%)		(%)		(%)	
Age in years							
Median (range)	45.5 (28–67)		45.5 (31–67)		45.0 (28–52)		0.26
Mean (SD)	46.08 (9.6)		47.5 (11.2)		43.9 (6.2)		0.26

Ethnicity							
Chinese	28	(70.0)	19	(67.9)	9	(32.1)	0.20
Other Asian	11	(27.5)	5	(45.5)	6	(54.5)	
Caucasian	1	(2.5)	0		1	(100)	

Mode of presentation							
Symptomatic tumours	29	(72.5)	18	(62.1)	11	(37.9)	0.67
Screen detected lesions	11	(27.5)	5	(45.5)	6	(54.5)	

Pathologic tumour size in mm							
Median (range)	20.0		18.5 (0–72)		20.0 (0–55)		
Mean (SD)	19.6 (14)^*∗*^		20.0 (14.9)		20.1 (12.22)		0.99
<20 mm (T1)	23	(57.5)	14	(60.9)	9	(39.1)	0.85
20–<50 mm (T2)	14	(35.0)	8	(57.1)	6	(42.9)	
>50 mm (T3)	2	(5.0)	1	(50.0)	1	(50.0)	
Skin and/or chest wall involved (T4)	1	(2.5)	0		1	(100)	

Stage at diagnosis							
0	5	(12.5)	4	(80.0)	1	(20.0)	0.06
I	13	(32.5)	9	(69.2)	4	(30.8)	
II	17	(42.5)	7	(41.2)	10	(58.8)	
III	5	(12.5)	4	(80.0)	1	(20.0)	
IV	0						

Histological type							
DCIS	5	(12.5)	4	(80.0)	1	(20.0)	0.21
Invasive ductal	30	(75.0)	19	(63.3)	11	(36.7)	
Invasive lobular	4	(10.0)	1	(25.0)	3	(75.0)	
Other invasive	1	(2.5)	0		1	(100)	

Grade							
DCIS	5	(12.5)	4	(80.0)	1	(20.0)	***0.02***
1	6	(15.0)	3	(50.0)	3	(50.0)	
2	13	(32.5)	4	(30.8)	9	(69.2)	
3	15	(37.5)	13	(86.7)	2	(13.3)	
Unknown	1	(2.5)			1	(100)	

Hormone-receptor status							
Positive	28	(70.0)	15	(53.6)	13	(46.4)	0.51
Negative	10	(25.0)	7	(70.0)	3	(30.0)	
Unknown	2	(5.0)	2	(100)	0		

Neoadjuvant medical therapy							
No	11	(27.5)	5	(45.5)	6	(54.5)	0.25
Yes	29	(72.5)	19	(65.5)	10	(34.5)	

Surgical procedure							
BCT	34	(85.0)	21	(87.5)^+^	13	(81.3)^+^	0.46
Mastectomy by need	5	(12.5)	2	(8.3)	3	(18.7)	
Mastectomy by choice	1	(2.5)	1	(4.2)			

Reoperations	4	(10.0)					
Axillary dissection	1	(2.5)	1				
Missed multicentric	3	(7.5)			3		

Recurrence							
Locoregional recurrence	1	(2.5)			1		
Distant disease/death	2	(5.0)	1		1		

Median follow-up (months)	59						
(range)	(43–75)						

5-year breast cancer-specific survival	95.7%		100%		87.5%		Log-rank test: 0.47

5-year disease-free survival	92.7%		100%		80.8%		Log-rank test: 0.52

MFMCBC: multifocal, multicentric breast cancer.

BCT: breast conservation treatment; SD: standard deviation.

^*∗*^Dimension of largest lesion.

^+^Percentage expressed as the number undergoing BCT in the MF or MC group, respectively.

**Table 2:** Patients' self-assessment and clinician's evaluation of cosmetic outcome.

	Patient's assessment			Clinician's assessment		
	Multifocal	Multicentric	Combined (%)	Multifocal	Multicentric	Combined (%)
Excellent: 5	5	6	(55.0)	3	5	(40.0)
Good: 4	4	1	(25.0)	7	1	(40.0)
Satisfactory: 3	4	0	(20.0)	1	1	(10.0)
Fair: 2	0	0		2	0	(10.0)
Poor: 1	0	0		0	0	
Total	13	7		13	7	
			*p* = 0.05			*p* = 0.13

Kappa value = 0.11.
